# Identifying hospitalization episodes of care among people with and without HIV in British Columbia, Canada

**DOI:** 10.23889/ijpds.v9i5.2549

**Published:** 2024-09-18

**Authors:** Scott Emerson, Taylor McLinden, Paul Sereda, Amanda Yonkman, Jason Trigg, Rolando Barrios, Robert Hogg

**Affiliations:** 1 BC Centre for Excellence in HIV/AIDS; 2 Simon Fraser University

## Background

Hospitalizations are a resource-intensive form of healthcare use, particularly for persons with chronic conditions such as those with HIV. Interhospital transfers typically appear as separate records in Canadian databases; misclassifying transfers as independent hospitalizations can bias key metrics such as readmission rates. We examined approaches of combining sequential, related records into hospitalization episodes of care (HEoCs) among persons with and without HIV (PWH; PWoH) in British Columbia (BC), Canada.

## Methods

BC hospitalization records (1992 to 2020) were sourced from the Comparative Outcomes and Service Utilization Trends (COAST) study, a data linkage that includes samples of PWH and PWoH. We constructed 8 HEoC definitions that varied by the: a) time gap between records, and b) transfer indication. Comparisons were informed by the proportion of multi-record HEoCs (mHEoCs; episodes with multiple hospitalization records) generated, and feasibility given data quality.

## Results

We analyzed 98,553 hospitalization records from 13,498 PWH, and 1,874,507 hospitalization records from 385,011 PWoH. Across the definitions, the proportion of mHEoCs varied from 2.46% to 5.27% for PWH and 2.73% to 4.18% for PWoH. Definitions requiring no transfer indication yielded the highest proportion of mHEoCs, whereas those requiring two-way agreement of hospital identifiers yielded the lowest proportion of mHEoCs. Patterns were comparable among PWH and PWoH. A pragmatic approach to defining HEoCs can be a reasonable option for general purposes – requiring at least one populated hospital identifier field, and ≤ 1 day gap between hospitalizations.

## Conclusions

Various approaches can be employed to combine sequential, related hospitalization records into HEoCs to help provide less biased estimates of hospitalization-related metrics.

